# Association of sagittal abdominal diameter with cardiovascular disease and cardiometabolic risk factors among US adults: A cross-sectional study

**DOI:** 10.1097/MD.0000000000044594

**Published:** 2025-09-19

**Authors:** Can Chen, Xiao Liang, Lingyan Fang, Hemanyun Bai, Kangling Ke, Haitao Huang

**Affiliations:** a Department of Cardiology, Second Affiliated Hospital of Guangdong Medical University, Zhanjiang, Guangdong Province, China; b Department of Ultrasound Medicine, Central People’s Hospital of Zhanjiang, Zhanjiang, Guangdong Province, China; c Department of Anesthesiology, First Affiliated Hospital of Guangdong Medical University, Zhanjiang, Guangdong Province, China.

**Keywords:** cardiometabolic risk factors, cardiovascular disease, NHANES, sagittal abdominal diameter, visceral fat

## Abstract

Obesity, a major cardiovascular disease (CVD) risk factor, can be assessed through sagittal abdominal diameter (SAD), though its link to CVD is less studied. This study investigates the relationship between SAD and CVD and compares the predictive ability of SAD, body mass index (BMI), and waist circumference (WC) for CVD in US adults. A cross-sectional study was conducted using data from the National Health and Nutrition Examination Survey (NHANES) 2011 to 2016. Restricted cubic splines and multivariable logistic regression were used to assess the association between SAD and CVD. The predictive performance of SAD, BMI, and WC was compared using receiver operating characteristic curve analysis. The study included 10,854 adults aged 20 years and older (representing 158.7 million; mean age 45.7 ± 16.1 years; 50.6% male, 66.3% non-Hispanic White). The weighted mean (95% confidence interval [CI]) of SAD was 22.6 (22.4–22.8) cm, and the prevalence of CVD was 7.0%. Higher SAD was associated with increased CVD prevalence (odds ratio [OR], 1.11; 95% CI, 1.08–1.13) after multivariate adjustment. Compared with participants with SAD < 19.4 cm, those with SAD > 25.4 cm had a significantly higher cardiovascular risk (OR, 1.87; 95% CI, 1.31–2.67). The area under the receiver operating characteristic curve for SAD (0.65 [0.64–0.65]) was the largest compared to BMI (0.57 [0.56–0.58]) and WC (0.63 [0.62–0.64]). Higher SAD was associated with more CVD events, and it demonstrated better clinical utility for predicting CVD risk compared with BMI and WC.

## 1. Introduction

Cardiovascular disease (CVD) is the leading cause of death worldwide, contributing to 20.5 million deaths in 2021.^[1]^ Obesity is a primary risk factor for CVD and leads to cardiovascular complications, which are influenced by both the amount and distribution of body fat.^[2,3]^ Based on distribution, body fat is classified into subcutaneous and visceral fat, with excessive visceral fat serving as an independent predictor of adverse cardiovascular outcomes.^[4]^

Traditionally, body mass index (BMI) and waist circumference (WC) are used to assess obesity. BMI measures general obesity but is a heterogeneous indicator that does not distinguish between muscle and fat or capture fat distribution.^[5]^ WC estimates abdominal fat and is often associated with metabolic disease and CVD. While WC is superior to BMI in predicting cardiovascular risk to some extent, it does not distinguish between subcutaneous and visceral fat, offering only a rough estimate of central obesity.

Sagittal abdominal diameter (SAD), also known as “abdominal height,” provides a more accurate estimate of visceral obesity, independent of overall fatness. In contrast, WC may be a better indicator of subcutaneous adipose tissue (SAT).^[6]^ This study aims to investigate the association between SAD and both CVD and various cardiometabolic risk factors using a nationally representative sample of US adults.

## 2. Methods

### 2.1. Study design and participants

**Figure 1.**
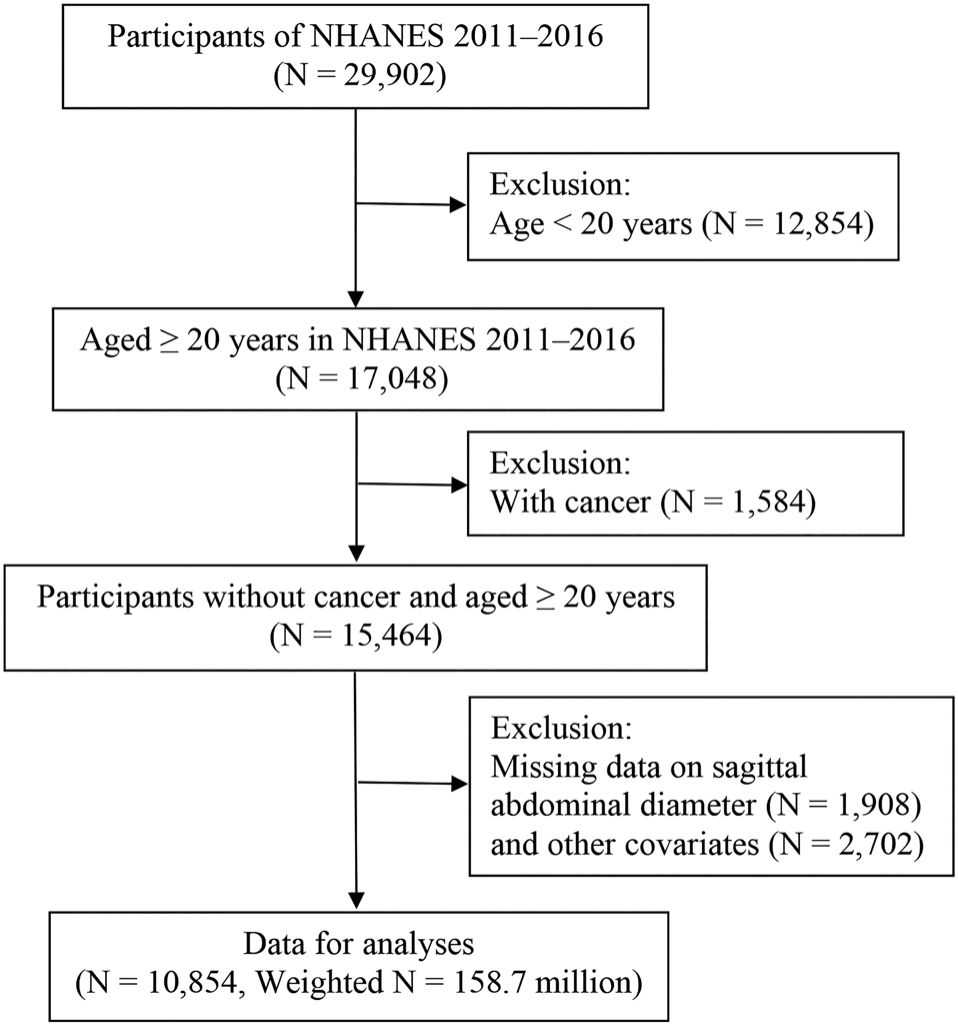
Flowchart of participant selection.

The present study used data from the NHANES, a large-scale, cross-sectional, nationally representative survey of the US population conducted by the NCHS and Centers for Disease Control and Prevention. Structured interview data and physical examination results were collected continuously and released in 2-year cycles. All participants provided written informed consent. During the 2011 to 2016 NHANES cycles, 17,048 participants aged ≥ 20 years, were interviewed. Exclusions were made for 1584 participants with a history of cancer, 1908 with missing data on SAD, and 2702 with incomplete data on CVD or covariates. A final sample of 10,854 participants was included, representing 158.7 million non-institutionalized US residents (Fig. 1).

### 2.2. Anthropometry

A Holtain-Kahn caliper was used to measure individuals’ SAD. Participants (excluding pregnant women and those weighing over 600 pounds [272.2 kg]) were positioned supine with feet flat and knees flexed. The iliac crest was located at the point where it intersected the midaxillary line, and a line was drawn perpendicular to the table on the uppermost lateral border of the ilium. The distance between the 2 caliper arms was measured at the end of exhalation by placing 1 arm on the back and the other on the abdomen. At least 2 measurements of SAD to the nearest 0.1 cm were conducted by trained health technicians, following standardized procedures from the NHANES Anthropometry Procedures Manual. Third and fourth measurements were necessary if the first and second measurements differed by more than 0.5 cm.

BMI was calculated as weight in kilograms divided by height in meters squared. WC was measured at the midpoint between the lower rib margin and the iliac crest using a tape measure, with participants standing. Anthropometric measurements are described in the NHANES Anthropometry Procedures Manual.

### 2.3. Ascertainment of CVD

CVD was determined based on participants’ responses to a standardized and validated medical history questionnaire, including congestive heart failure (CHF), coronary heart disease (CHD), myocardial infarction (MI, also called heart attack), angina, and stroke. Specifically, a positive answer to the question “Has a doctor or other health professional ever told you had CHF, CHD, heart attack, angina, or stroke?” was defined as having CVD.^[7,8]^ Additionally, CHD was re-defined as a composite of self-reported CHD, MI, and angina. CVD was the primary outcome in this analysis, while CHF, CHD, and stroke were the secondary outcomes.

### 2.4. Covariates

Since many covariates affect CVD, we included some common variables according to their degree of correlation with independent variables and referred to other research. Data regarding age, sex, race/ethnicity, education level, poverty index, smoking status, leisure-time physical activity, and comorbidities were collected from household interviews. Blood pressure (BP) and alcohol consumption data were obtained from the mobile examination center. Chronic kidney disease (CKD) was defined as an eGFR < 60 ml/min/1.73 m^2^ or a urinary albumin-to-creatinine ratio ≥ 30 mg/g, based on the KDIGO 2021 clinical practice guidelines.^[9]^ Race/ethnicity was classified as non-Hispanic White, non-Hispanic Black, Mexican American, or other. Education level was classified as < high school, high school, or > high school. The poverty index (the ratio of family income to the federal poverty threshold) was classified as < 1.30, 1.30 to 3.49, or ≥ 3.50. Smoking status was categorized as never, current, or former. Alcohol consumption was categorized as heavy drinker (≥ 4 drinks/day for males, ≥ 3 drinks/day for females, or binge drinking ≥ 5 days/month), moderate drinker (≥ 2 drinks/day for males, ≥ 1 drink/day for females, or binge drinking ≥ 2 days/month), and nondrinker. Leisure-time physical activity was categorized as “inactive” (no reported physical activity data), “somewhat active” (< 500 MET min/week), and “active” (≥ 500 MET min/week).^[10]^ Diabetes was defined as a self-reported diagnosis of diabetes, use of hypoglycemic medication or hemoglobin A_1c_ (HbA_1c_) ≥ 6.5%. Hypertension was defined as a self-reported diagnosis of hypertension, use of oral antihypertensive medication, or mean BP determinations (systolic BP ≥ 140 mm Hg and/or diastolic BP ≥ 90 mm Hg) in the mobile examination center.

Moreover, cardiometabolic biomarkers, including plasma glucose, insulin, HbA_1c_, total cholesterol (TC), triglyceride (TG), low-density lipoprotein cholesterol (LDL-C), high-density lipoprotein cholesterol (HDL-C), and high sensitivity C-reactive protein (hs-CRP), were obtained from laboratory examination (hs-CRP data are only available in the NHANES 2015–2016). Homeostasis model assessment of insulin resistance (HOMA-IR) was calculated based on the method described by Matthews.^[11]^ The visceral adiposity index (VAI), an adiposity index of visceral fat function associated with cardiometabolic risk that represents the level of visceral adipose tissue (VAT), was calculated based on the WC, TG, and HDL-C levels.^[12]^

### 2.5. Statistical analysis

Sample weights, clustering, and stratification were incorporated in all analyses because of the complex sampling design of the NHANES. Participants were stratified into quartiles based on SAD: < 19.4, 19.4 to 22.2, 22.3 to 25.4, and > 25.4 cm. Data were reported as weighted mean (standard deviation) for continuous variables and count (weighted percentage) for categorical variables. The mean levels of cardiometabolic biomarkers and their standard errors were reported for each SAD category. A generalized linear model was applied to examine the associations of SAD as a continuous variable. Pearson correlation coefficients were calculated to evaluate the correlation between VAT and SAD, WC, and BMI. Multivariable logistic regression estimated odds ratios and 95% confidence intervals for CVD and its components (CHF, CHD, and stroke) across SAD quartiles. Model 1 was adjusted for age, sex, and race/ethnicity. Model 2 was additionally adjusted for education level, poverty index, smoking status, alcohol consumption, and leisure-time physical activity. Model 3 was adjusted for model 2 plus CKD, hypertension, and diabetes. Linear trend was tested by assigning a median value to each category as a continuous variable. Furthermore, we applied logistic regression based on a restricted cubic spline with 4 knots to examine the dose-response relationship between SAD and CVD.

Stratified analyses were also performed according to age (≤ 60 or > 60 years), sex (male or female), race/ethnicity (White or nonwhite), education level, poverty index, smoking status, alcohol consumption, leisure-time physical activity, comorbidity (none or any event of CKD, hypertension or diabetes). The *P*-values for the product terms between SADs and stratification variables were used to estimate the significance of the interactions. Sensitivity analysis was conducted to test the robustness of the identified associations and to examine whether cardiometabolic biomarkers could explain the observed association. Plasma glucose, insulin, HOMA-IR, HbA_1c_, TC, HDL-C, LDL-C, TG, and hs-CRP levels were further adjusted in the sensitivity analyses (available data ranged from 3535 to 10,854). Finally, the receiver operating characteristic curve was established, and the area under the curve (AUC) was calculated to compare the predictive ability of SAD, BMI, and WC for CVD. Statistical analyses were performed using the “survey” package in R 4.1.2. A 2-sided *P* <.05 was considered statistically significant.

## 3. Results

### 3.1. Baseline characteristics of study participants

Baseline characteristics across SAD quartiles are summarized in Table 1. Among the 10,854 adults (mean age, 45.7 ± 16.1 years; 50.6% males), the weighted mean (95% CI) of SAD was 22.6 (22.4–22.8) cm. The prevalence of CVD, CHF, CHD, and stroke was observed in 7.0%, 1.9%, 4.9%, and 2.2% of participants, respectively, with corresponding counts of 930, 281, 636, and 315. Participants with higher SAD were more likely to be older, non-Hispanic Black, former smokers, nondrinkers, and physically inactive, but less likely to have higher levels of education, be physically active, or be moderate drinkers.

**Table 1:** Characteristics of participants according to quartiles of sagittal abdominal diameter.

	Sagittal abdominal diameter (cm)				
	< 19.4	19.4–22.2	22.3–25.4	> 25.4	Total
Characteristics	(n = 2634)	(n = 2697)	(n = 2709)	(n = 2814)	(N = 10,854)
Age, mean ± SE, year	39.2 ± 0.6	45.5 ± 0.4	48.8 ± 0.4	49.4 ± 0.5	45.7 ± 0.4
Male	1056 (37.3)	1419 (52.0)	1505 (56.5)	1514 (56.8)	5494 (50.6)
Race/ethnicity					
Non-Hispanic White	1006 (67.0)	969 (65.6)	1013 (66.0)	1117 (66.4)	4105 (66.3)
Non-Hispanic Black	418 (8.1)	531 (9.7)	600 (10.6)	836 (14.7)	2385 (10.8)
Mexican American	243 (6.3)	403 (9.2)	462 (10.5)	419 (9.1)	1527 (8.7)
Other Race	967 (18.6)	794 (15.5)	634 (12.9)	442 (9.8)	2837 (14.2)
Education level					
Less than high school	439 (11.7)	572 (14.2)	624 (15.7)	626 (14.7)	2261 (14.0)
High school	480 (16.6)	555 (19.9)	637 (22.4)	711 (25.4)	2383 (21.1)
More than high school	1715 (71.7)	1570 (65.9)	1448 (61.9)	1477 (59.9)	6210 (64.9)
Poverty index					
< 1.30	812 (22.2)	831 (21.5)	939 (23.0)	1020 (24.1)	3602 (22.7)
1.30–3.49	898 (31.4)	987 (36.0)	970 (34.8)	1082 (38.6)	3937 (35.2)
≥ 3.50	924 (46.4)	879 (42.5)	800 (42.2)	712 (37.4)	3315 (42.1)
Smoking status					
Never smoke	1707 (63.4)	1572 (58.0)	1489 (55.9)	1463 (50.1)	6231 (56.9)
Current smoke	553 (20.3)	553 (20.4)	548 (18.8)	573 (19.2)	2227 (19.7)
Former smoke	374 (16.4)	572 (21.6)	672 (25.3)	778 (30.7)	2396 (23.5)
Alcohol consumption					
Nondrinker	686 (18.9)	765 (23.1)	862 (25.3)	969 (29.4)	3282 (24.1)
Moderate drinker	1390 (57.8)	1395 (55.2)	1283 (52.7)	1241 (48.4)	5309 (53.5)
Heavy drinker	558 (23.4)	537 (21.7)	564 (22.1)	604 (22.2)	2263 (22.3)
Physical activity					
Inactive	979 (30.9)	1182 (37.7)	1377 (47.6)	1658 (55.6)	5196 (42.9)
Somewhat active	369 (13.9)	398 (16.3)	398 (15.3)	400 (16.7)	1565 (15.5)
Active	1286 (55.2)	1117 (46.0)	934 (37.0)	756 (27.7)	4093 (41.6)
BMI, mean ± SE, kg/m^2^	22.5 ± 0.1	26.5 ± 0.1	30.1 ± 0.1	37.2 ± 0.2	29.0 ± 0.1
WC, mean ± SE, cm	81.2 ± 0.2	93.3 ± 0.2	103.3 ± 0.2	120.1 ± 0.4	99.4 ± 0.3
Medical condition					
Hypertension	508 (16.5)	910 (29.2)	1240 (42.0)	1675 (55.5)	4333 (35.7)
Diabetes	106 (2.5)	282 (6.5)	459 (12.4)	857 (25.5)	1704 (11.7)
Chronic kidney disease	273 (9.4)	352 (10.3)	444 (12.9)	656 (19.3)	1725 (13.0)
Congestive heart failure	24 (0.6)	44 (1.2)	73 (1.9)	140 (3.9)	281 (1.9)
Coronary heart disease	61 (1.6)	119 (3.7)	185 (5.4)	271 (8.9)	636 (4.9)
Stroke	41 (1.3)	63 (1.8)	91 (2.6)	120 (3.4)	315 (2.2)
Cardiovascular disease	93 (2.6)	177 (5.3)	267 (7.9)	393 (12.3)	930 (7.0)

BMI = body mass index, SE = standard Error, WC = waist circumference.

Values are numbers (weighted percentages), unless specified as mean (SE).

### 3.2. Association between SAD and cardiometabolic markers

As presented in Table 2, cardiometabolic biomarkers’ geometric means and their standard error were reported for each SAD category and the generalized linear model was applied to examine the associations of SAD, as a continuous variable. Higher SAD was significantly associated with higher levels of glucose, insulin, HOMA-IR, HbA_1c_, TG, and hs-CRP and lower levels of HDL-C (all *P* trend <.001). The weighted Pearson correlation coefficients of glucose, insulin, HOMA-IR, HbA_1c_, TG, HDL-C, and hs-CRP were 0.27, 0.40, 0.34, 0.29, 0.20, −0.35, and 0.29, respectively.

**Table 2:** Association of cardiometabolic marker concentrations with sagittal abdominal diameter among US adults, aged ≥ 20 years.

	Sagittal abdominal diameter (cm)*				Per/unit increment		
Cardiometabolic markers	< 19.4	19.4–22.2	22.3–25.4	> 25.4	*β* (95%CI)†	*P*_trend_	*Coef.*
Glucose (n = 5236), mg/dL	95.6 ± 0.7	101.9 ± 0.6	106.1 ± 0.6	121.8 ± 1.5	0.90 (0.71–1.10)	<.001	0.27
Insulin (n = 5143), pmol/L	39.5 ± 1.3	55.2 ± 1.3	77.7 ± 2.1	139.9 ± 5.2	8.65 (7.64–9.66)	<.001	0.40
HOMA-IR (n = 5140)	1.59 ± 0.06	2.34 ± 0.06	3.49 ± 0.11	7.61 ± 0.37	0.45 (0.39–0.52)	<.001	0.34
HbA1c (n = 10,810), %	5.29 ± 0.01	5.47 ± 0.02	5.67 ± 0.03	6.04 ± 0.04	0.02 (0.02–0.03)	<.001	0.29
TC (n = 10,805), mg/dL	184.1 ± 1.1	194.8 ± 1.1	198.1 ± 1.0	192.7 ± 1.0	0.74 (0.50–0.98)	<.001	0.05
TG (n = 5136), mg/dL	82.3 ± 1.9	112.3 ± 2.4	139.4 ± 4.8	158.8 ± 5.5	4.85 (3.88–5.82)	<.001	0.20
LDL-C (n = 5052), mg/dL	104.1 ± 1.2	115.9 ± 1.1	120.4 ± 1.3	113.9 ± 1.2	1.05 (0.83–1.28)	<.001	0.09
HDL-C (n = 10,805), mg/dL	62.9 ± 0.6	54.6 ± 0.6	50.3 ± 0.4	45.8 ± 0.4	−1.28 (−1.40 to −1.16)	<.001	-0.35
hs-CRP (n = 3535), mg/L	1.6 ± 0.2	2.7 ± 0.2	3.7 ± 0.2	6.3 ± 0.4	0.44 (0.36–0.52)	<.001	0.29

Coef. = Pearson correlation coefficient, HOMA-IR = homeostasis model assessment of insulin resistance, TC = total cholesterol, TG = triglyceride, LDL-C = low-density lipoprotein cholesterol, HDL-C = high-density lipoprotein cholesterol, hs-CRP = high sensitivity C-reactive protein.

† The increase in cardiometabolic marker concentration caused by a 1-unit increase in sagittal abdominal diameter was estimated using a weighted linear model with adjustments for age, sex, race/ethnicity, education level, poverty index, smoking status, alcohol consumption, physical activity, chronic kidney disease, hypertension, and diabetes.

* Means (standard error) of cardiometabolic marker concentrations are given by quartiles of sagittal abdominal diameter.

### 3.3. Association between SAD and VAI

VAI was calculated among 6135 participants (3064 [50.1%] males) with available WC, TG, and LDL-C data. The weighted means (95% CI) of VAI were 2.01 (1.88–2.14) in males and 1.90 (1.76–2.04) in females. Pearson correlation coefficients between VAI and SAD, WC, and BMI were 0.220 (0.225 in males, 0.214 in females), 0.215 (0.216 in males, 0.213 in females), and 0.172 (0.199 in males, 0.155 in females), respectively (All *P* <.001).

### 3.4. Association between SAD and CVD

**Figure 2.**
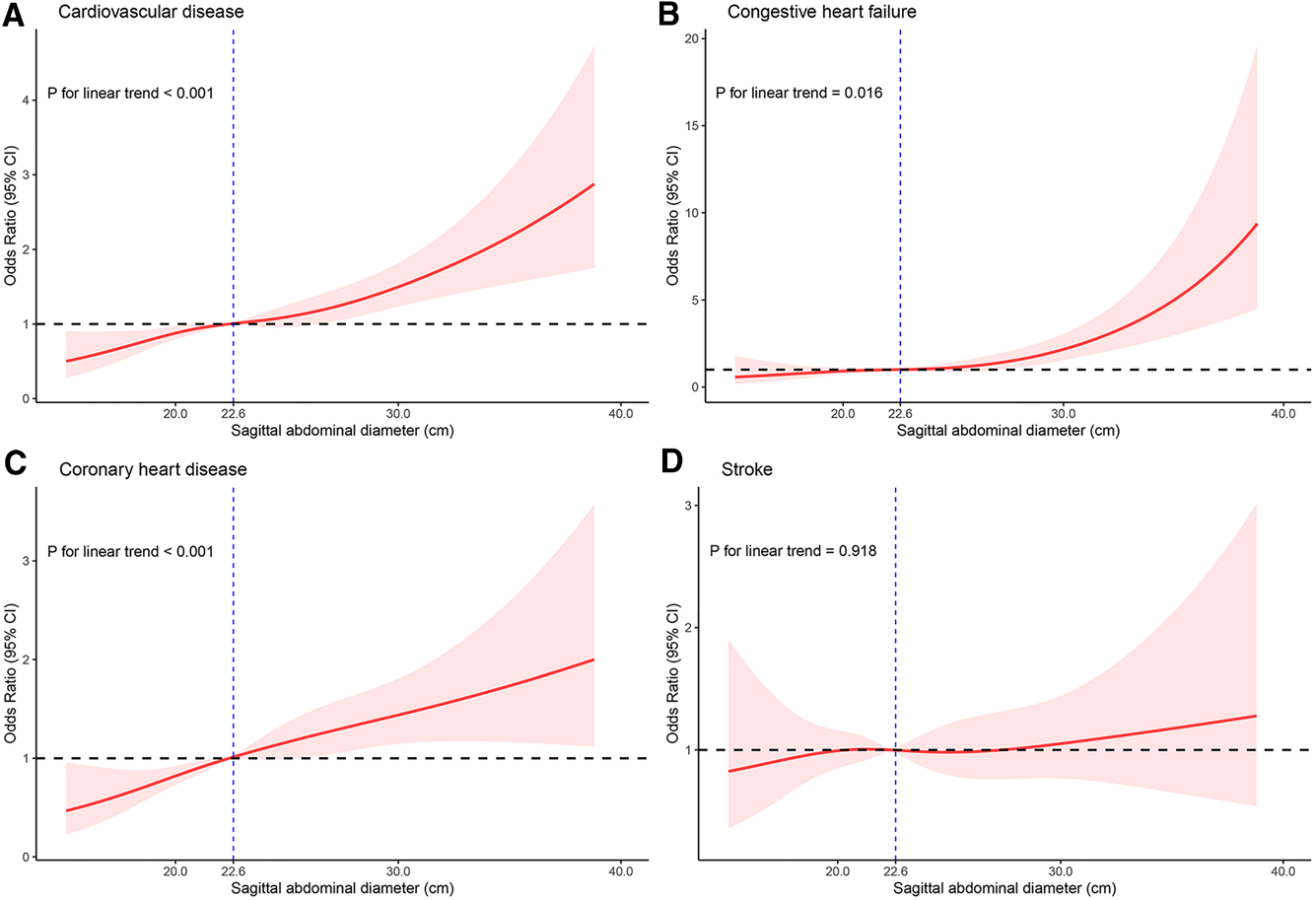
The dose-response analysis between sagittal abdominal diameter and cardiovascular disease with restricted cubic splines. The solid lines indicate multivariate-adjusted ORs, and the dashed lines indicate the 95% CIs derived from restricted cubic spline regression. Four knots were used to examine a dose-response relationship between sagittal abdominal diameter and cardiovascular disease (including congestive heart failure, coronary heart disease, and stroke). The logistic regression was adjusted for age, sex, race/ethnicity, education level, poverty index, smoking status, alcohol consumption, physical activity, chronic kidney disease, hypertension, and diabetes. CIs = confidence intervals, ORs = odds ratios.

A significantly positive dose-response relationship was observed between SAD and the risk of CVD, CHF, and CHD (all *P* for linear trend <.05; Fig. 2). Participants in the highest SAD quartile had higher odds of CVD (OR, 1.87; 95% CI: 1.31–2.67), CHF (OR, 2.38; 95% CI: 1.18–4.79), and CHD (OR, 2.12; 95% CI: 1.40–3.21) compared to those in the lowest quartile, with no significant association for stroke (OR, 0.94; 95% CI: 0.55–1.60) after adjusting for confounders (Table 3). After multivariate adjustment, each 1-unit increment in SAD was associated with a 6% increased risk of CVD (11% for CHF and 6% for CHD). Although the odds ratios decreased with additional adjustments, the results remained consistent. No linear relationship was found between SAD and stroke (*P* for linear trend = .918).

**Table 3:** Odds ratios (95% CIs) for cardiovascular disease according to sagittal abdominal diameter levels among US adults in the NHANES 2011 to 2016.

	Sagittal abdominal diameter (cm)				*P* for	Per/unit increment
	< 19.4	19.4–22.2	22.3–25.4	> 25.4	trend	OR (95% CI)
Cardiovascular disease						
No. (%) cases	93 (2.63)	177 (5.34)	267 (7.92)	393 (12.29)	–	–
Model 1	Ref.	1.38 (0.95–2.00)	1.76 (1.27–2.44)	2.95 (2.18–3.99)	<.001	1.11 (1.08–1.13)
Model 2	Ref.	1.32 (0.88–1.98)	1.74 (1.20–2.51)	2.71 (1.96–3.74)	<.001	1.10 (1.08–1.13)
Model 3	Ref.	1.21 (0.80–1.85)	1.47 (0.99–2.19)	1.87 (1.31–2.67)	<.001	1.06 (1.03–1.09)
Congestive heart failure						
No. (%) cases	24 (0.59)	44 (1.23)	73 (1.87)	140 (3.88)	–	–
Model 1	Ref.	1.42 (0.74–2.72)	1.82 (1.00–3.30)	3.98 (2.31–6.88)	<.001	1.16 (1.11–1.22)
Model 2	Ref.	1.37 (0.67–2.77)	1.77 (0.96–3.27)	3.63 (1.97–6.68)	<.001	1.16 (1.10–1.22)
Model 3	Ref.	1.23 (0.61–2.51)	1.46 (0.76–2.80)	2.38 (1.18–4.79)	.016	1.11 (1.05–1.18)
Coronary heart disease						
No. (%) cases	61 (1.62)	119 (3.75)	185 (5.45)	271 (8.88)	–	–
Model 1	1.00	1.53 (0.98–2.37)	1.87 (1.27–2.75)	3.25 (2.27–4.65)	<.001	1.10 (1.08–1.13)
Model 2	1.00	1.48 (0.95–2.31)	1.89 (1.26–2.84)	3.08 (2.12–4.45)	<.001	1.10 (1.08–1.13)
Model 3	1.00	1.36 (0.86–2.15)	1.59 (1.02–2.48)	2.12 (1.40–3.21)	<.001	1.06 (1.03–1.09)
Stroke						
No. (%) cases	41 (1.28)	63 (1.79)	91 (2.57)	120 (3.37)	–	–
Model 1	1.00	0.95 (0.51–1.78)	1.16 (0.67–1.99)	1.51 (0.94–2.41)	.049	1.05 (1.01–1.09)
Model 2	1.00	0.90 (0.45–1.79)	1.10 (0.61–1.99)	1.32 (0.81–2.16)	.11	1.04 (1.01–1.08)
Model 3	1.00	0.83 (0.41–1.67)	0.94 (0.50–1.78)	0.94 (0.55–1.60)	.918	1.01 (0.98–1.04)

CI = confident interval, NHANES = National Health and Nutrition Examination Survey.

No. (%) cases showed as numbers (weighted percentages).

Model 1: adjusted for age, sex, race/ethnicity;

Model 2: adjusted for Model 1 plus education level, poverty index, smoking status, alcohol consumption, leisure-time physical activity;

Model 3: adjusted for Model 2 plus chronic kidney disease, hypertension, and diabetes. The statistical significance of a linear trend was assessed using the weighted regression and modeling the midpoint of each group.

### 3.5. Subgroup and sensitivity analysis

Consistent results were observed across various subgroup, including age, sex, race/ethnicity, education level, poverty index, smoking status, alcohol consumption, leisure-time physical activity, and comorbidity (Table S1, Supplemental Digital Content, https://links.lww.com/MD/P986). No significant interactions were detected between SAD and these stratifying variables (all *P* for interaction >.05). Additionally, when further adjusted for glucose, insulin, HOMA-IR, HbA_1c_, TC, HDL-C, LDL-C, TG, or hs-CRP levels, the results did not materially change, although the association between SAD and CVD risk was slightly attenuated (Table S2, Supplemental Digital Content, https://links.lww.com/MD/P986).

### 3.6. Diagnostic efficacy of anthropometric measurements in CVD

**Figure 3.**
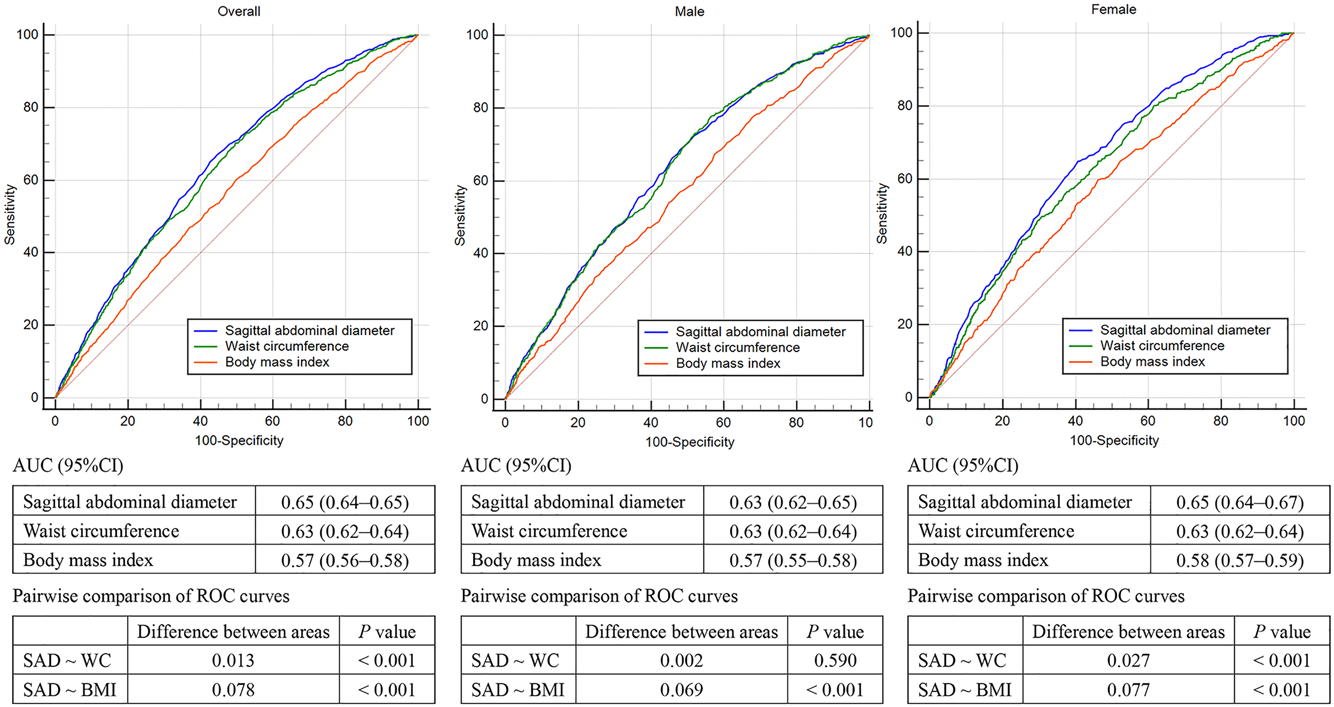
Comparison of ROC curves of the anthropometric indices for diagnosis efficacy of predicting cardiovascular disease by sex. ROC = receiver operating characteristic.

The area under the receiver operating characteristic curve of SAD (AUC, 0.65; 95% CI: 0.64–0.65) was higher than that of WC (AUC, 0.63; 95% CI: 0.62–0.64) and BMI (AUC, 0.57; 95% CI: 0.56–0.58) and pairwise comparison between different areas between SAD with WC and BMI were statistically significant (Fig. 3). When stratified by sex, we observed consistent results in females, however, the AUC for SAD was close to WC in males. The optimal cutoff value of SAD, WC, and BMI for the prediction of obesity-related CVD was 22.7 cm, 96.8 cm and 27.8 kg/m^2^, respectively (Table S3, Supplemental Digital Content, https://links.lww.com/MD/P986). The optimal cutoff of SAD was similar in males (22.3 cm) and females (22.8 cm); however, the cutoff values of WC (95.7 cm for males, 104.4 cm for females) and BMI (26.6 kg/m^2^ for males, 28.9 kg/m^2^ for females) vary widely between the sexes.

## 4. Discussion

In this nationally representative study, we found that a higher SAD was associated with increased odds of CVD, CHF, and CHD, showing a linear dose-response relationship after multivariable adjustment. Findings remained consistent across subgroups stratified by age, sex, race/ethnicity, education level, smoking, alcohol consumption, physical activity, and comorbidity, with no significant interactions detected. Sensitivity analyses further confirmed the robustness of these associations. SAD correlated more strongly with VAT (via VAI) than WC or BMI, suggesting its reliability in representing visceral fat. Pearson correlation analysis also demonstrated positive associations between SAD and blood glucose, insulin resistance, TG, and hs-CRP, supporting its link with cardiometabolic risk factors.^[13]^ Furthermore, SAD outperformed BMI (but not WC) in predicting CVD, particularly in females, and showed comparable predictive efficiency to WC in males. Optimal SAD cutoff values were similar between sexes. In summary, elevated SAD was associated with an increased risk of CVD. Its predictive capability for obesity-related CVD events was comparable to, if not superior to, those of WC and BMI.

Abdominal fat is divided into shallow SAT and deep adipose tissue (namely, VAT). Epidemiological studies have shown that VAT is an independent marker of morbidity and mortality, whereas abdominal SAT accumulation is a weaker indicator of cardiovascular risk.^[14]^ Increased VAT contributes to ectopic lipid deposition and metabolic disturbances through dysregulated adipokine secretion and excessive fatty acid release.^[15]^ Additionally, VAT is strongly associated with insulin resistance and inflammatory dysregulation and produces more CVD- and metabolism-related cytokines than SAT.^[16,17]^ Similarly, SAD was linked to insulin resistance, elevated hs-CRP, hypertriglyceridemia, and reduced HDL-C. Interestingly, SAD showed only a weak association with TC and LDL-C, yet its correlation with CVD risk increased after adjusting for “bad” lipids. This discrepancy is because our study did not distinguish between large and small LDL, but “normal” LDL cholesterol.^[18]^ We hypothesize that high SAD is linked to increased small dense LDL and reduced large LDL; however, further research is needed to confirm this association. This phenomenon also partly explains the low HDL level in patients with visceral obesity and highlights the importance of differentiating LDL particle size and density when assessing lipid profiles.

Obesity is a major risk factor for CVD in the US. However, 41.3% of overweight and 9.7% of obese adults do not perceive themselves as overweight.^[5]^ With rising obesity rates, CVD incidence will continue to increase unless more effective prevention and treatment strategies are implemented. A key step in addressing this issue is developing a simple, clinically applicable tool to distinguish VAT from SAT and improve self-awareness. Anthropometric measures such as BMI, WC, and waist-to-hip ratio are commonly used to assess adiposity. BMI is widely applied due to its simplicity but fails to differentiate between fat and muscle mass or fat distribution.^[18]^ Its relationship with CVD mortality follows a J-shaped curve, complicating clinical interpretation.^[19]^ Moreover, VAT levels vary significantly among individuals with the same BMI, and the obesity paradox further challenges its reliability as a sole predictor.^[20,21]^ WC provides better insight into fat distribution and independently predicts CVD risk. However, its cutoff values vary by race, sex, and age, limiting its universal applicability.^[22]^ In females, hormonal differences affect VAT accumulation, with rapid changes after menopause.^[23]^ WC correlates more strongly with SAT than VAT, yet SAT shows no clear linear relationship with obesity-related risk factors.^[24–27]^ While guidelines recommend WC as a CVD risk marker, inconsistent cutoff values reduce its clinical utility.

SAD is a simple yet effective measure of obesity and VAT, making it the best anthropometric index for predicting abdominal obesity.^[28]^ Compared to BMI and WC, SAD better represents VAT while minimizing the influence of SAT. Studies show that SAT is only weakly or insignificantly associated with CVD risk.^[24,29–31]^ WC quantifies abdominal size but fails to differentiate between SAT and VAT, potentially leading to inaccurate CVD risk assessments. SAD can accurately evaluate VAT because its measurement is performed with the subject in the supine position, whereas SAT tends to slide to the sides of the body because of its higher malleability. VAT is a more rigid tissue and does not slide, which makes SAD better for VAT analysis. Yim et al demonstrated that SAD had the strongest correlation with VAT across age, sex, and obesity levels compared to BMI and WC.^[6]^ BMI cannot distinguish between lean mass and fat mass, limiting its ability to reflect VAT accurately.^[32]^ Although CT and MRI are gold standards for VAT measurement, their cost and radiation exposure make them impractical for routine screening. In contrast, SAD is quick, affordable, and easy to perform. Notably, its threshold for CVD risk is nearly identical in males and females, enhancing its clinical applicability. Given its advantages, SAD holds promise for both epidemiological studies and clinical risk assessment.

While SAD is a promising anthropometric measure, it has certain limitations. First, its measurement requires specialized tools, making it less convenient than BMI, WC, or hip circumference in fast-paced clinical settings. Second, SAD primarily reflects visceral fat, overlooking adipose distribution in other body regions, which may limit its use in comprehensive obesity and metabolic assessments. Additionally, the lack of standardized measurement protocols may lead to variability across studies and clinical practices, reducing its comparability and applicability. Although research supports the association between SAD and chronic disease risk, further large-scale studies are needed to validate its reliability across diverse populations and conditions. As with any emerging metric, clinical adoption takes time. However, with ongoing research and technological advancements, SAD is expected to gain increasing recognition and application in clinical practice.

Our study has several limitations. First, its observational nature prevents causal inferences. Second, self-reported CVD diagnoses and medication use may be affected by recall bias. Third, we used NHANES 2011 to 2016, the most recent cycles with standardized SAD data, as later cycles do not include such measurements; however, the age of the data may limit temporal generalizability. Fourth, we could not directly assess the correlation between VAT and SAD, BMI, or WC using CT/MR, as NHANES lacks imaging data. Fifth, the absence of hip circumference measurements precluded the calculation of waist-to-hip ratio, limiting comparisons with SAD. Finally, as our study is based on the US population, its applicability to non-US populations requires further investigation.

## 5. Conclusion

Elevated SAD predicts a higher risk of CVD events and better reflects visceral fat than WC and BMI. Given its strong correlation with insulin resistance, inflammation, and lipid disorders, SAD may serve as a superior predictor of CVD risk. However, as this study is cross-sectional, further real-world studies are needed to validate these findings.

## Acknowledgments

Thanks to Zhang Jing (Second Department of Infectious Disease, Shanghai Fifth People’s Hospital, Fudan University) for his work on the NHANES database. His outstanding work, nhanesR package and webpage, makes it easier for us to explore NHANES database.

## Author contributions

**Conceptualization:** Haitao Huang.

**Data curation:** Haitao Huang.

**Formal analysis:** Haitao Huang.

**Funding acquisition:** Can Chen.

**Investigation:** Haitao Huang.

**Methodology:** Can Chen, Haitao Huang.

**Project administration:** Can Chen.

**Supervision:** Xiao Liang, Haitao Huang.

**Visualization:** Haitao Huang.

**Writing – original draft:** Hemanyun Bai, Kangling Ke.

**Writing – review & editing:** Can Chen, Xiao Liang, Lingyan Fang, Haitao Huang.

## Supplementary Material


