# Supplementary material for: Association of sagittal abdominal diameter with cardiovascular disease and cardiometabolic risk factors among US adults: A cross-sectional study
Source: Medicine (Baltimore). 2025 Sep 19;104(38):e44594. doi: 10.1097/MD.0000000000044594 (PMC12459509; doi:10.1097/MD.0000000000044594)
Supplement: Supplementary file 1 [file medi-104-e44594-s001.docx]

**Supplemental Table S1.** Stratified analyses of the associations between sagittal abdominal diameter and cardiovascular disease, NHANES 2011–2016

| Stratification group | Sagittal abdominal diameter (cm) | | | | | *P* for interaction |
| --- | --- | --- | --- | --- | --- | --- |
|  | <19.4 | 19.4–22.2 | 22.3–25.4 | >25.4 | *P* trend |  |
| Age (years) |  |  |  |  |  | 0.854 |
| ≤60 | 1.00 | 1.35 (0.70–2.61) | 1.64 (0.91–2.97) | 1.79 (1.01–3.18) | 0.038 |  |
| >60 | 1.00 | 1.24 (0.78–1.97) | 1.54 (0.94–2.53) | 1.85 (1.10–3.13) | 0.02 |  |
| Sex |  |  |  |  |  | 0.324 |
| Female | 1.00 | 1.45 (0.90–2.35) | 1.32 (0.83–2.12) | 1.88 (1.18–3.00) | 0.023 |  |
| Male | 1.00 | 0.92 (0.51–1.64) | 1.43 (0.76–2.69) | 1.59 (0.89–2.83) | 0.028 |  |
| Race/ethnicity |  |  |  |  |  | 0.804 |
| White | 1.00 | 1.19 (0.67–2.12) | 1.52 (0.89–2.58) | 1.94 (1.19–3.16) | 0.006 |  |
| Non-White | 1.00 | 1.18 (0.70–1.99) | 1.34 (0.86–2.08) | 1.54 (1.03–2.29) | 0.024 |  |
| Education level |  |  |  |  |  | 0.406 |
| <High school | 1.00 | 1.58 (0.65–3.80) | 1.31 (0.73–2.37) | 2.51 (1.31–4.79) | 0.004 |  |
| High School | 1.00 | 1.27 (0.50–3.24) | 2.06 (0.83–5.08) | 2.42 (1.08–5.39) | 0.002 |  |
| >High school | 1.00 | 1.06 (0.64–1.77) | 1.32 (0.82–2.14) | 1.43 (0.87–2.36) | 0.118 |  |
| Poverty index |  |  |  |  |  | 0.386 |
| <1.30 | 1.00 | 0.80 (0.39–1.65) | 0.97 (0.55–1.70) | 1.35 (0.77–2.39) | 0.061 |  |
| 1.30–3.49 | 1.00 | 1.74 (0.97–3.12) | 2.34 (1.16–4.72) | 2.41 (1.28–4.53) | 0.018 |  |
| ≥3.50 | 1.00 | 1.20 (0.52–2.74) | 1.49 (0.77–2.87) | 1.96 (0.96–3.99) | 0.043 |  |
| Smoking status |  |  |  |  |  | 0.167 |
| Never smoker | 1.00 | 0.80 (0.43–1.48) | 1.22 (0.70–2.13) | 1.33 (0.75–2.34) | 0.099 |  |
| Current smoker | 1.00 | 1.96 (0.86–4.47) | 2.34 (1.14–4.81) | 2.53 (1.51–4.25) | 0.002 |  |
| Former smoker | 1.00 | 1.45 (0.68–3.08) | 1.50 (0.69–3.27) | 2.34 (1.08–5.10) | 0.016 |  |
| Alcohol consumption |  |  |  |  |  | 0.733 |
| Nondrinker | 1.00 | 1.08 (0.57–2.06) | 1.04 (0.57–1.91) | 1.55 (0.81–2.96) | 0.101 |  |
| Moderate drinker | 1.00 | 1.18 (0.62–2.23) | 1.76 (0.99–3.12) | 1.84 (1.15–2.95) | 0.007 |  |
| Heavy drinker | 1.00 | 1.48 (0.49–4.41) | 1.54 (0.65–3.63) | 2.47 (1.03–5.92) | 0.023 |  |
| Physical activity |  |  |  |  |  | 0.101 |
| Inactive | 1.00 | 1.30 (0.83–2.05) | 1.42 (0.90–2.22) | 1.88 (1.28–2.74) | 0.006 |  |
| Somewhat active | 1.00 | 1.34 (0.34–5.38) | 4.23 (1.38–12.99) | 3.93 (1.10–13.97) | 0.002 |  |
| Active | 1.00 | 1.07 (0.60–1.91) | 1.21 (0.62–2.37) | 1.39 (0.75–2.57) | 0.26 |  |
| Comorbidity |  |  |  |  |  | 0.051 |
| None | 1.00 | 0.68 (0.32–1.45) | 0.82 (0.42–1.60) | 1.28 (0.66–2.50) | 0.403 |  |
| Any | 1.00 | 1.70 (1.07–2.69) | 2.08 (1.37–3.15) | 2.87 (1.86–4.42) | <0.001 |  |

NHANES = National Health and Nutrition Examination Survey.

Analysis was adjusted for age, sex, race/ethnicity, education level, poverty index, smoking status, alcohol consumption, physical activity, chronic kidney disease, hypertension, and diabetes, with exception of stratifying factors. Comorbidities included chronic kidney disease, hypertension, and diabetes.

**Supplemental Table S2.** Odds ratios of cardiovascular disease according to sagittal abdominal diameter with further adjustment of cardiometabolic markers

|  | Sagittal abdominal diameter (cm) | | | | *P* for |
| --- | --- | --- | --- | --- | --- |
|  | <19.4 | 19.4–22.2 | 22.3–25.4 | >25.4 | trend |
| Participants with data of glucose (n=5236) | | |  |  |  |
| Model 1 | 1.00 | 1.70 (0.96–2.98) | 1.68 (1.00–2.82) | 2.98 (1.92–4.62) | <0.001 |
| Model 2 | 1.00 | 1.45 (0.77–2.73) | 1.41 (0.78–2.55) | 2.08 (1.28–3.36) | <0.001 |
| Model 2+Glucose | 1.00 | 1.44 (0.76–2.70) | 1.40 (0.77–2.54) | 1.99 (1.22–3.26) | 0.003 |
| Participants with data of insulin (n=5143) | | |  |  |  |
| Model 1 | 1.00 | 1.73 (0.98–3.06) | 1.65 (0.97–2.81) | 2.99 (1.93–4.62) | <0.001 |
| Model 2 | 1.00 | 1.47 (0.78–2.77) | 1.38 (0.75–2.54) | 2.05 (1.26–3.33) | 0.004 |
| Model 2+Insulin | 1.00 | 1.46 (0.78–2.76) | 1.36 (0.74–2.50) | 1.96 (1.20–3.22) | 0.011 |
| Participants with data of HOMA-IR (n=5140) | | |  |  |  |
| Model 1 | 1.00 | 1.73 (0.98–3.06) | 1.65 (0.97–2.81) | 2.98 (1.93–4.62) | <0.001 |
| Model 2 | 1.00 | 1.47 (0.78–2.77) | 1.38 (0.75–2.53) | 2.05 (1.26–3.33) | 0.004 |
| Model 2+HOMA-IR | 1.00 | 1.47 (0.78–2.76) | 1.36 (0.74–2.51) | 1.98 (1.21–3.23) | 0.009 |
| Participants with data of HbA1c (n=10810) | | |  |  |  |
| Model 1 | 1.00 | 1.37 (0.94–1.99) | 1.73 (1.25–2.41) | 2.95 (2.18–3.98) | <0.001 |
| Model 2 | 1.00 | 1.21 (0.79–1.84) | 1.47 (0.99–2.18) | 1.89 (1.32–2.69) | <0.001 |
| Model 2+HbA_1c_ | 1.00 | 1.21 (0.79–1.83) | 1.46 (0.98–2.17) | 1.87 (1.31–2.67) | 0.001 |
| Participants with data of TC and HDL-C (n=10805) | | |  |  |  |
| Model 1 | 1.00 | 1.38 (0.95–2.01) | 1.74 (1.25–2.42) | 2.97 (2.20–4.01) | <0.001 |
| Model 2 | 1.00 | 1.21 (0.80–1.84) | 1.47 (0.99–2.18) | 1.89 (1.33–2.69) | <0.001 |
| Model 2+TC | 1.00 | 1.29 (0.85–1.96) | 1.57 (1.05–2.35) | 2.00 (1.41–2.82) | <0.001 |
| Model 2+HDL-C | 1.00 | 1.10 (0.72–1.67) | 1.26 (0.83–1.91) | 1.55 (1.09–2.21) | 0.011 |
| Participants with data of LDL-C (n=5052) | | |  |  |  |
| Model 1 | 1.00 | 1.73 (0.97–3.11) | 1.63 (0.94–2.82) | 2.93 (1.84–4.67) | <0.001 |
| Model 2 | 1.00 | 1.42 (0.74–2.73) | 1.34 (0.72–2.52) | 2.02 (1.22–3.34) | 0.005 |
| Model 2+LDL-C | 1.00 | 1.56 (0.82–2.96) | 1.52 (0.81–2.86) | 2.28 (1.40–3.72) | 0.001 |
| Participants with data of TG (n=5136) | | |  |  |  |
| Model 1 | 1.00 | 1.73 (0.97–3.09) | 1.67 (0.97–2.87) | 2.91 (1.83–4.62) | <0.001 |
| Model 2 | 1.00 | 1.42 (0.74–2.73) | 1.37 (0.74–2.55) | 1.98 (1.20–3.28) | 0.006 |
| Model 2+TG | 1.00 | 1.42 (0.73–2.75) | 1.37 (0.71–2.61) | 1.97 (1.16–3.34) | 0.008 |
| Participants with data of hs-CRP (n=3535) | | |  |  |  |
| Model 1 | 1.00 | 1.20 (0.57–2.51) | 1.84 (0.80–4.23) | 3.37 (1.78–6.40) | <0.001 |
| Model 2 | 1.00 | 0.98 (0.47–2.03) | 1.44 (0.62–3.36) | 2.40 (1.26–4.58) | 0.005 |
| Model 2+hs-CRP | 1.00 | 0.96 (0.46–2.02) | 1.41 (0.61–3.29) | 2.30 (1.20–4.43) | 0.006 |

HOMA-IR = homeostasis model assessment of insulin resistance; TC = total cholesterol; HDL-C = high density lipoprotein cholesterol; LDL-C = low-density lipoprotein cholesterol; TG = triglyceride; hs-CRP = high sensitivity C-reactive protein.

Model 1: adjusted for age, sex, race/ethnicity;

Model 2: adjusted for Model 1 plus education level, poverty index, smoking status, alcohol consumption, leisure time physical activity, chronic kidney disease, hypertension, and diabetes.

| **Supplemental Table S3.** Diagnostic efficacy of anthropometric measures and predicting patients with cardiovascular disease stratified by sex. | | | | | | | |
| --- | --- | --- | --- | --- | --- | --- | --- |
|  | AUC | Cut-off | PPV (%) | NPV (%) | Sensitivity (%) | Specificity (%) | Youden index |
| Overall |  |  |  |  |  |  |  |
| SAD | 0.65 | 22.7 | 12.1 | 94.8 | 67.0 | 55.4 | 0.22 |
| WC | 0.63 | 96.8 | 11.5 | 94.8 | 70.2 | 50.3 | 0.20 |
| BMI | 0.57 | 27.8 | 10.0 | 93.2 | 60.4 | 49.8 | 0.10 |
| Male |  |  |  |  |  |  |  |
| SAD | 0.63 | 22.3 | 12.9 | 94.3 | 72.5 | 48.2 | 0.21 |
| WC | 0.63 | 95.7 | 12.7 | 94.8 | 77.5 | 43.8 | 0.21 |
| BMI | 0.57 | 26.6 | 11.0 | 92.5 | 67.4 | 42.4 | 0.10 |
| Female |  |  |  |  |  |  |  |
| SAD | 0.65 | 22.8 | 11.1 | 95.6 | 64.9 | 59.2 | 0.24 |
| WC | 0.63 | 104.4 | 11.4 | 94.6 | 48.6 | 70.4 | 0.19 |
| BMI | 0.58 | 28.9 | 9.2 | 94.5 | 59.8 | 53.7 | 0.14 |

AUC = the area under the receiver operating characteristic curve; PPV = positive predictive value; NPV = negative predictive value; SAD = sagittal abdominal diameter; WC = waist circumference; BMI = body mass index.
